# Lateral Intercostal Artery Perforator Flap for Axillary Breast Reconstruction in a Previously Irradiated and Dissected Field

**DOI:** 10.1055/s-0046-1817012

**Published:** 2026-04-15

**Authors:** Niki K. Patel, Joshua Henderson, Halil Safak Uygur

**Affiliations:** 1 Division of Plastic, Reconstructive, and Hand Surgery, Department of Surgery, West Virginia University School of Medicine, Morgantown, West Virginia, United States

**Keywords:** LICAP flap, axillary reconstruction, oncoplastic surgery

## Abstract

Reconstruction of axillary defects after oncologic resection is particularly challenging in patients with prior axillary dissection and radiation, as traditional thoracodorsal-based flaps may be unreliable. We present a case of a 59-year-old woman with recurrent left axillary invasive ductal carcinoma, 18 years after lumpectomy, axillary dissection, chemotherapy, and radiation. En bloc resection involving the pectoralis minor and serratus anterior created a 6 × 5 × 2 cm defect with exposed rib. A lateral intercostal artery perforator (LICAP) flap was used for reconstruction, based on a dominant perforator at the sixth intercostal space identified by Doppler and confirmed intraoperatively with indocyanine green angiography. The flap was de-epithelialized, tunneled to the axilla, and the donor site closed primarily. The patient achieved complete healing without necrosis or contracture and maintained full shoulder mobility after adjuvant chemoradiation. The LICAP flap offers a safe, reliable option for axillary reconstruction in previously treated fields.

## Introduction

Local perforator flaps have become a cornerstone in oncoplastic reconstruction due to their favorable donor site profile, preservation of muscle function, and reliable vascularity. 1 2 3 4 The lateral intercostal artery perforator (LICAP) flap is most commonly described for lateral breast volume replacement, but also has potential applications in other regions of the chest wall. 1 3 5 6 7 Its use in axillary reconstruction, particularly in previously irradiated or dissected fields, remains under-recognized. In high-risk surgical settings, careful flap selection is essential to optimize healing, preserve function, and support adjuvant therapy. We present a case demonstrating the successful use of a LICAP flap for immediate axillary reconstruction following oncologic resection in a previously treated field.

## Case Report

A 59-year-old woman with a history of left breast invasive ductal carcinoma was initially treated 18 years ago with lumpectomy, axillary lymph node dissection, adjuvant chemotherapy, radiation, and endocrine therapy. In early 2024, routine surveillance imaging identified a recurrence in the left axilla involving the pectoralis minor and serratus anterior muscles. Following neoadjuvant chemotherapy, the patient underwent a low axillary en bloc resection of the recurrent tumor.

The resultant defect measured 6 × 5 × 2 cm and included exposed rib. Given the history of axillary dissection and radiation, the reconstructive challenge was to provide durable, well-vascularized coverage while preserving mobility and minimizing the risk of future contracture and wound complications—particularly in anticipation of additional adjuvant radiation.

**Fig. 1:**
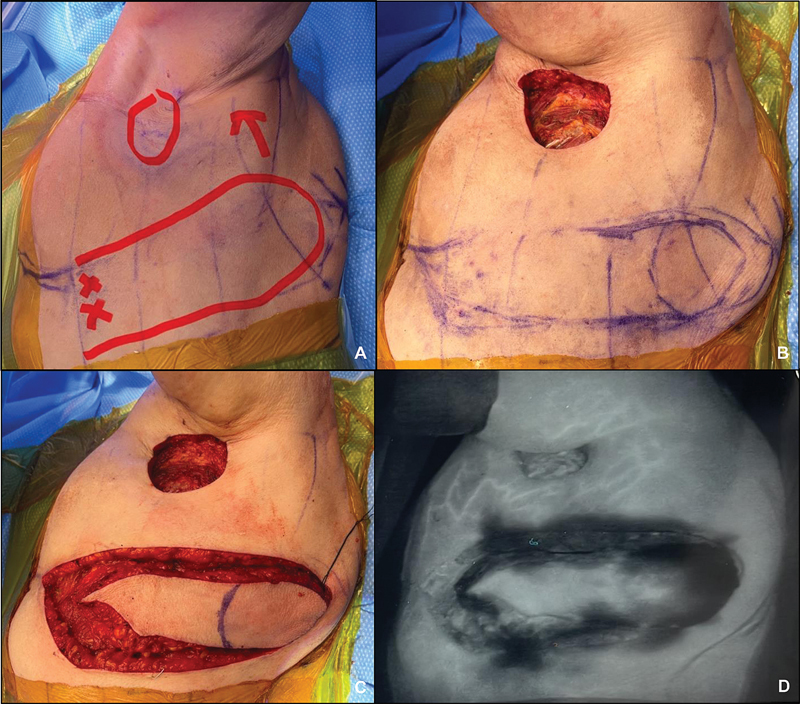
( **A** ) The fasciocutaneous flap was designed in a propeller fashion, extending laterally and posteriorly to provide enough reach. ( **B** ) Final axillary wound status post low axillary en bloc resection. ( **C** ) The fasciocutaneous flap was elevated in a subfascial plane, preserving the vascular supply while allowing flexibility in inset. ( **D** ) Indocyanine green (ICG) SPY imaging was used to confirm flap perfusion. The distal tip of the flap was trimmed after observing lower perfusion at the tip.

A LICAP flap was selected due to concerns for thoracodorsal artery compromise and the need to avoid irradiated vascular territories, including the thoracodorsal artery perforators. Using handheld Doppler, the dominant lateral intercostal artery perforator was identified at the level of the sixth intercostal space along the lateral chest wall, just anterior to the anterior border of the latissimus dorsi muscle and outside the previously irradiated field. 6 7 8 A propeller-style fasciocutaneous flap was designed extending laterally and posteriorly, and elevated in a subfascial plane. The perforator was isolated during dissection. Intraoperative indocyanine green (ICG) angiography with SPY imaging confirmed adequate perfusion, and the distal tip was trimmed due to relatively lower signal intensity ( Fig. 1 ).

**Fig. 2:**
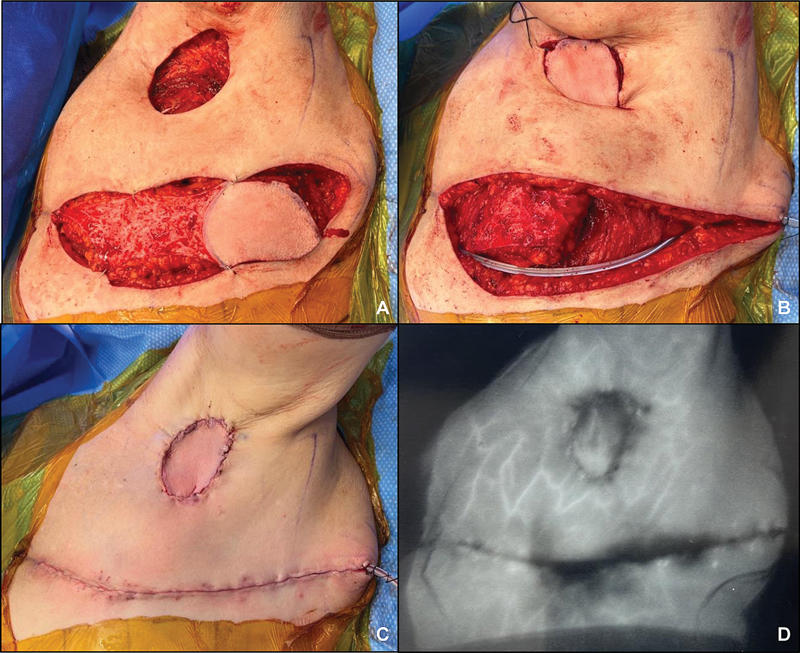
( **A, B** ) Once confirmed, the flap was de-epithelialized and tunneled into the axilla. ( **C** ) The flap was inset, and the donor site was closed primarily without tension. ( **D** ) Indocyanine green (ICG) SPY imaging was used to confirm flap perfusion after closure.

The flap was de-epithelialized and tunneled into the axilla to fill the defect. The donor site was closed primarily without tension, with ICG SPY imaging again used to confirm perfusion following inset ( Fig. 2 ).

**Fig. 3:**
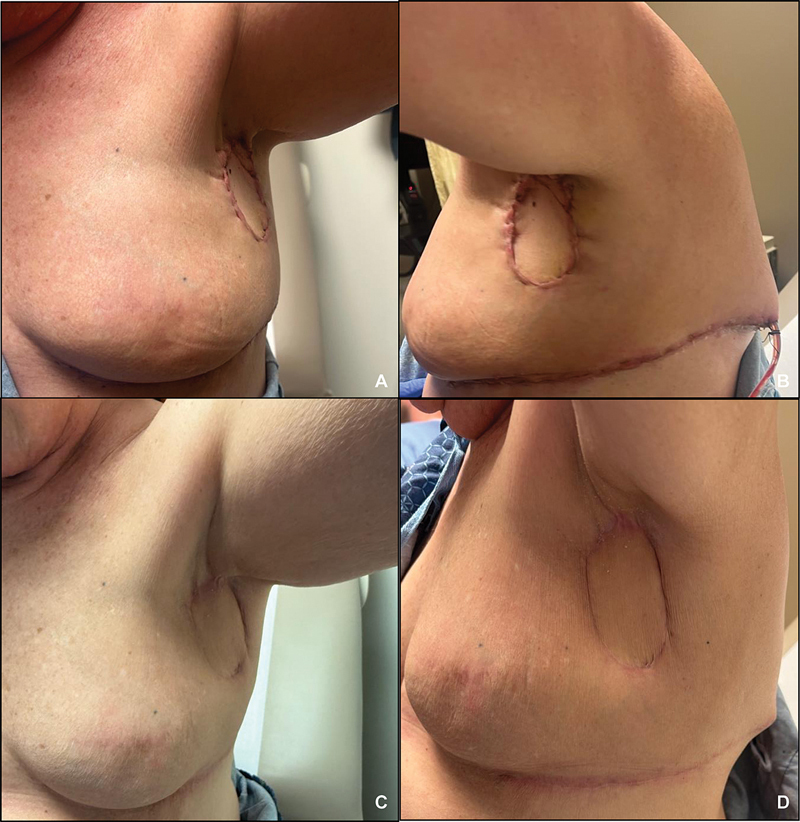
( **A, B** ) The patient at 1-week postoperative visit. ( **C, D** ) The patient at 7 months postoperatively.

Postoperatively, the patient demonstrated excellent flap viability with no necrosis or wound complications. At 3 weeks, there was no evidence of axillary contracture and full shoulder mobility was preserved. At 6-month follow-up, following completion of chemotherapy and re-irradiation, the flap remained stable with no functional deficits ( Fig. 3 ).

## Discussion

Reconstruction of axillary defects in the setting of prior surgery and radiation requires thoughtful selection of technique and tissue source. Traditional local flaps based on the thoracodorsal system—such as thoracodorsal artery perforator (TDAP) or latissimus dorsi flaps—may not be reliable when the vascular supply has been compromised by previous axillary dissection or radiation. 5 9 10

The latissimus dorsi flap, in particular, is widely considered a workhorse for salvage breast and chest wall reconstruction due to its robust vascularity and large arc of rotation. 9 However, even when uninjured, the thoracodorsal vessels may be scarred or at risk in previously operated fields. 9 In this case, we chose to preserve the latissimus dorsi as a reserve option for future salvage procedures, should postoperative healing complications or disease recurrence occur—a strategy supported by reconstructive algorithms in complex axillary dissections.

TDAP flaps are also frequently used in breast and axillary reconstruction due to their pliability and reliable vascularity. 5 10 However, these flaps depend on the thoracodorsal pedicle and may not be ideal in patients with prior axillary intervention. Favorable outcomes have been reported using local perforator flaps in delayed and secondary breast reconstruction, though success depends heavily on the integrity of available perforators. 5 In direct comparisons, LICAP flaps have demonstrated reconstructive outcomes similar to TDAP flaps, while offering the potential for reduced dissection and operative time. 10

The LICAP flap has been described for volume replacement in small to moderate breast defects and is increasingly recognized as a reliable option in oncoplastic reconstruction. 1 2 4 7 However, its broader utility in complex reconstructive scenarios—such as axillary reconstruction in previously irradiated or dissected fields—remains underutilized. In this case, its anatomy made it an advantageous choice: the dominant perforator, located at the sixth intercostal space, 7 lay outside both the previous dissection and radiation fields, allowing transfer of healthy, well-vascularized tissue into a compromised zone.

Although LICAP flaps do not offer the same robust vascular supply as axial flaps, their perforators can be reliable when properly identified and evaluated. 7 8 Anatomically, LICAPs have been described to lie approximately 1.0 to 3.5 cm anterior to the anterior border of the latissimus dorsi muscle, with the dominant perforator most frequently encountered at the level of the sixth intercostal space. 7 8 Based on this vascular anatomy, the flap can be designed along the lateral thoracic wall to address small to moderate defects, with posterior extension toward the posterior thoracic region when additional reach is required. 7 8 Reported experience suggests that flap length may approach approximately 20 to 25 cm, with widths of up to 12 cm allowing for primary donor-site closure. 7 8 However, extension toward the anterior or posterior midline results in increasing dependence on random-pattern perfusion and should therefore be undertaken with caution. 7 8 In the present case, handheld Doppler and intraoperative ICG angiography were used to confirm perfusion and guide flap design and inset.

Additionally, skin grafting was not a viable option in this case due to the presence of exposed rib and the inherent mobility of the axilla. Even if the wound had not involved bone, the risk of contracture and poor durability—particularly with anticipated postoperative radiation—would have made skin grafting suboptimal. A flap was clearly necessary to provide durable coverage, maintain axillary mobility, and minimize the risk of wound breakdown in a high-risk field.

This case highlights the utility of the LICAP flap as a versatile and underused option in complex reconstructions. Its ability to provide pliable, well-vascularized tissue from outside previously treated fields makes it particularly well suited to high-risk cases where traditional flaps are contraindicated or best reserved.

## Conclusion

The LICAP flap is a reliable and technically accessible option for axillary reconstruction, particularly in previously irradiated or dissected surgical fields. It provides durable coverage, minimizes donor site morbidity, and plays a critical role in preserving shoulder function and enabling adjuvant therapy. Although traditionally used for partial breast reconstruction, this case demonstrates its broader reconstructive potential and supports greater consideration of the LICAP flap in high-risk oncoplastic procedures.
